# Les complications thromboemboliques chez les enfants au cours de COVID-19

**DOI:** 10.11604/pamj.2021.40.137.31979

**Published:** 2021-11-04

**Authors:** Amal El Ouarradi, Ilham Bensahi, Insaf Alaammari, Amal Haoudar, Nezha Dini, Mohamed Sabry

**Affiliations:** 1 Cardiology Department, Mohammed VI University of Health Sciences, Mohammed VI International University Hospital, Casablanca, Morocco,; 2 Pediatric Department, Mohammed VI University Of Health Sciences, Mohammed VI International University Hospital, Casablanca, Morocco,; 3 Reanimation Department, Mohammed VI University Of Health Sciences, Cheikh Khalifa Hospital, Casablanca, Morocco,; 4 Pediatrics Department, Pedagogy and Research Unit, Mohammed V University of Medicine, Rabat, Morocco

**Keywords:** COVID-19, embolie pulmonaire, thrombose veineuse, enfant, anticoagulation

## Aux éditeurs de Pan African Medical Journal

Les complications thromboemboliques chez les enfants émergent dans l´ère de COVID-19, on rapporte cette lettre pour attirer l´attention sur ces complications, leurs incidences, le diagnostic et la prise en charge et surtout l´intérêt de l´anticoagulation préventive chez cette population fragile. Il existe de nombreuses différences entre les enfants et les adultes en ce qui concerne la prévalence, la gravité et les complications de l´infection COVID-19. Le pourcentage d'enfants touchés par la COVID-19 est beaucoup plus faible que celui des adultes et ils ont tendance à présenter des symptômes plus légers et une morbidité et une mortalité significativement plus faibles [1,2]. Bien que l'on ait beaucoup appris sur les taux et les risques de complications thromboemboliques (TE) chez les adultes, on en sait relativement peu sur ce problème dans la population pédiatrique. L'incidence globale de la COVID-19 et de la thrombose est nettement inférieure chez les enfants, ce qui a ajouté des défis à l'étude de cette condition dans ce groupe d'âge [3]. Après avoir rapporté le cas d´une jeune patiente de 14 ans ayant présenté une embolie pulmonaire grave dans les suites d´infection COVID-19 [4], voilà le cas d´un enfant encore plus jeune, de 5 ans, sans ATCD pathologique particulier, ayant présenté une détresse respiratoire dans les suites d´une infection COVID-19 avec un taux des D-dimere élevé, et un syndrome inflammatoire, et au scanner thoracique, une embolie pulmonaire massive bilatérale, avec retentissement sur les cavités droites à l´échocardiographie, et hypertension pulmonaire à 50 mmHg.

Des processus multifactoriels sont susceptibles de contribuer à la maladie thromboembolique veineuse et à l'immunothrombose dans le cadre de COVID-19: les anomalies microvasculaires distinctives de COVID-19 comprennent l'inflammation endothéliale, la perturbation des jonctions intercellulaires et la formation de microthrombus. Une coagulopathie distincte associée à la COVID-19, ainsi qu'une augmentation des cytokines et une activation des plaquettes, de l'endothélium et du complément, se produisent dans la COVID-19, ce qui est plus fréquent lorsque la maladie s'aggrave. Ce milieu pro-inflammatoire peut entraîner une immunothrombose, un mécanisme de défense de l'hôte qui peut devenir déréglé, conduisant à la formation excessive de thrombus à médiation immunologique qui affectent principalement la microvascularisation [5]. En plus, les enfants et les adolescents sont particulièrement touchés par un syndrome hyperinflammatoire post-infectieux après une infection par le SARS-CoV-2, appelé syndrome inflammatoire multisystémique chez l'enfant (SIM-E). Les patients atteints de SIM-E sont également à risque de thrombose [6]. Le syndrome inflammatoire multisystémique est défini selon l´OMS [7] par la survenue chez un enfant entre 0 et 19 ans d´une fièvre plus de 3 jours ET au moins deux des cinq éléments suivants: a) une éruption cutanée ou conjonctivite bilatérale non purulente ou inflammation cutanéo-muqueuse. b) Une hypotension ou choc. c) Des caractéristiques de dysfonctionnement myocardique, péricardite, valvulite ou anomalies coronaires (résultats d'écho, troponine/BNP élevée). d) Preuve de coagulopathie (PT ou PTT prolongé, D-Dimer élevé). e) Problèmes gastro-intestinaux aigus (diarrhée, vomissements ou douleurs abdominales). Associé à la présence de marqueurs d'inflammation élevés (ESR, CRP, Procalcitonine) sans autre cause microbienne évidente d'inflammation. Associé à une preuve de la présence de COVID-19 (RT-PCR, test antigénique ou sérologie positive), ou contact probable avec des patients atteints de COVID-19.

Le développement d'une complication TE avec une infection à COVID-19 a été associé à de mauvais résultats chez les enfants, comme cela a été décrit chez les adultes. Chez les patients pédiatriques, le taux de mortalité était de 28% avec complication TE contre 1% sans complication TE, et la plupart de ceux qui sont décédés avaient des comorbidités importantes. Chez les adultes atteints de COVID-19, l'estimation globale de la mortalité avec complications TE est de 23% contre 13% sans complications TE [8]. L'un des défis de la gestion du risque thrombotique dans la population pédiatrique est de déterminer qui est le plus susceptible de bénéficier d'une prophylaxie anticoagulante. Les taux de complications TE étant beaucoup plus faibles chez les enfants que chez les adultes, une approche globale utilisant la thromboprophylaxie pour tous les patients atteints de COVID-19 dans ce groupe d'âge peut exposer inutilement les patients à faible risque à des saignements. Un certain nombre de facteurs de risque TE ont été trouvés dans des études pédiatriques, y compris l'âge avancé (≥12 ans), ATCD de cancer. Ces facteurs, en combinaison avec la gravité de la maladie, l'assistance ventilatoire et l'élévation des D-dimères, pourraient être utilisés pour la stratification du risque et instaurer une thromboprophylaxie à base d´héparine de bas poids moléculaire ou d´héparine non fractionnée en cas d´insuffisance rénale [9].

## Conclusion

Les complications TE dans l´ère de COVID-19 reste redoutable chez les enfants comme chez l´adulte, d'où la nécessité d'une vigilance accrue face à cette complication. Une dose de prophylaxie efficace et une surveillance étroite sont recommandées chez les enfants atteints de COVID-19 et ayant des facteurs de risques TE.
